# Withaferin A ameliorates ovarian cancer-induced cachexia and proinflammatory signaling

**DOI:** 10.1186/s13048-019-0586-1

**Published:** 2019-11-25

**Authors:** Alex R. Straughn, Sham S. Kakar

**Affiliations:** 1 James Graham Brown Cancer Center, University of Louisville, Louisville, KY 40202 USA; 2 Department of Physiology, University of Louisville, School of Medicine, 500 South Floyd Street, Louisville, KY 40202 USA

**Keywords:** Cachexia, Cancer, Ovarian Cancer, Skeletal muscle, NF-κB, Inflammation, Cytokine

## Abstract

**Background:**

Ovarian cancer is the fifth leading cause of cancer-related deaths amongst women in the United States. Cachexia is the primary cause of death in approximately 30% of cancer patients, and is often evidenced in ovarian cancer patients. We tested the steroidal lactone Withaferin A to examine if it could ameliorate ovarian cancer-induced cachexia.

**Methods:**

Six-week-old severely immunodeficient female mice were xenografted with the ovarian cancer cell line A2780 followed by treatment with Withaferin A or vehicle. Changes in functional grip strength were assessed on a weekly basis. Postmortem, H&E staining was performed on skeletal muscle sections and immunofluorescent immunohistochemistry was performed on skeletal muscle and tumor sections. The levels of NF-κB-related proinflammatory cytokines were assessed in the xenografted tumors and in resident host skeletal muscle.

**Results:**

Xenografting of the A2780 cell line resulted in a significant rate of mortality, which was attenuated by a therapeutic dosage of Withaferin A. Mice that received vehicle treatment following xenografting exhibited functional muscle decline over the course of the study. The therapeutic dosage Withaferin A treatment attenuated this reduction in grip strength, whereas the supratherapeutic dosage of Withaferin A was found to be toxic/lethal and demonstrated a further decline in functional muscle strength and an increased rate of mortality on par with vehicle treatment. At a histological level, the vehicle treated tumor-bearing mice exhibited a profound reduction in myofibrillar cross-sectional area compared to the vehicle treated tumor-free control group. The atrophic changes induced by the xenografted tumor were significantly ameliorated by treatment with Withaferin A. The combination of functional muscle weakening and induction of myofibrillar atrophy corroborate a cachectic phenotype, which was functionally rescued by Withaferin A. Further, treatment completely abolished the slow-to-fast myofiber type conversion observed in the settings of cancer-induced cachexia. In both host resident skeletal muscle and the xenografted tumors, we report an increase in NF-κB-related proinflammatory cytokines that was reversed by Withaferin A treatment. Finally, we demonstrated that Withaferin A significantly downregulates cytosolic and nuclear levels of phospho-p65, the active canonical NF-κB transcription factor, in xenografted tumors.

**Conclusions:**

Cumulatively, our results demonstrate a previously overlooked role of Withaferin A in a xenograft model of ovarian cancer. We propose mechanisms by which Withaferin A reduces NF-κB-dependent pro-inflammatory cytokine production leading to an attenuation of the cachectic phenotype in an i.p. xenograft model of ovarian cancer.

## Introduction

Ovarian cancer is the most lethal gynecological malignancy and is the fifth leading cause of cancer-related deaths amongst women in the United States [1]. The early stages of ovarian cancer usually do not produce noticeable symptoms, therefore it is diagnosed at a late stage, where the cancer cells have already disseminated into the peritoneal cavity [2]. First-line therapy for late stage ovarian cancer is cytoreductive surgery followed by treatment with the platinum-based antineoplastic agent cisplatin or a combination of the taxane drug paclitaxel and carboplatin (a different platinum-based antineoplastic drug) [3, 4]. Current first-line therapy shows a high response rate initially, however, approximately 70% of patients relapse after a few rounds of treatment as a result of development of cisplatin resistance [5, 6].

Ovarian cancer is often associated with a complex metabolic syndrome, termed cachexia [7, 8]. Cachexia is a multifactorial disorder that is primarily characterized by a significant loss and/or weakening of skeletal muscle [9, 10]. It sometimes involves the loss of adipose tissue, but it is usually to a lesser extent than the loss of skeletal muscle [9]. Depending on the oncological setting, cachexia is exhibited in up to 80% of cancer patients and is the primary cause of mortality in up to 30% of cancer patients [9, 10]. Conflicting reports exist as to whether or not skeletal muscle atrophy occurs to a significant extent in the settings of ovarian cancer, but the hallmark of muscle weakening is consistently evidenced [11, 12]. Cachexia is highly correlated with a poor clinical prognosis, a decrease in quality of life, and a tolerance to antineoplastic agents [9, 10, 13]. In the context of ovarian cancer, cachexia usually accompanies the development of ascites and the onset of chemotherapeutic resistance [14]. Currently, there is no therapeutic treatment for cachexia as chemotherapeutic agents used to treat the primary disease often intensify the cachectic phenotype [15].

The steroidal lactone Withaferin A (WFA) is a purified extract from the *Withania somnifera* plant, also known as winter cherry or Ashwagandha. It is known for its anti-inflammatory properties and inhibitory effects on cell proliferation and invasion of ovarian cancer [16–20]. In the ovarian cancer cell lines SKOV3 and CAOV3, published reports have demonstrated that WFA induces cell cycle arrest and the induction of apoptosis, partially through targeting Notch signaling [21]. Previous work from our group has demonstrated that WFA leads to autophagic cell death in the ovarian cancer cell line A2780 through a rampant increase in reactive oxygen species production and subsequent DNA damage [16]. WFA is also notable due to its capability of targeting and inducing cell death of cancer stem cells, which are frequently spared by traditional chemotherapeutic agents [18]. Several studies performed by our group [16–20] and others [22–27] have investigated various effects of WFA under different oncological paradigms, but none of the published reports have considered the potential therapeutic application of WFA in the context of ovarian cancer-induced cachexia. Further, to the best of the authors’ knowledge, no clinical trial is currently investigating Withaferin A for its usage in ovarian cancer or sequela.

In the present study, we sought to establish whether or not WFA has an effect on cancer-induced cachexia and the associated weakening of skeletal muscle utilizing the ovarian surface epithelium cancer cell line A2780. As discussed below, WFA ameliorated gross body changes associated with ovarian cancer and cancer cachexia. Additionally, we report that treatment with WFA results in functional improvement of muscle strength and ameliorates myofiber-level changes caused by cachexia in our xenograft model. In resident host tissue and xenografted tumor, we demonstrate that WFA targets the canonical NF-κB pathway. To the best of our knowledge, this is the first report describing a novel role of WFA in ameliorating cancer-induced cachexia.

## Materials and methods

### Cell line

The A2780 ovarian epithelial cancer cell line was maintained in Roswell Park Memorial Institute (RPMI) Medium-1640 supplemented with: 10% Fetal Bovine Serum (FBS, Hyclone), 100 U/ml Penicillin, and 10 μg/ml Streptomycin. Cells were cultured in a humidified atmosphere of 5% CO_2_ at 37 °C, and the medium was changed every 48 h as described previously [18].

### Generation of tumor in mice

Six-week old female NOD.Cg-*Prkdc*^*scid*^ *Il2rg*^*tm1Wjl*^/SzJ (NSG, Jackson Lab Strain # 005557) immunodeficient mice were randomly assigned to one of three tumor-bearing groups or a tumor-free control group (5 animals/group), similar to as described in our previous study [20]. Tumor-bearing groups received an i.p. injection of 1.0 × 10^6^ low passage A2780 cells suspended in 100 μl sterile PBS. Control group received i.p. injection of 100 μl sterile PBS alone. After an initial refractory period of 8 days, the mice received an i.p. injection of vehicle (10% Dimethyl Sulfoxide, 90% Glycerol Trioctanoate) or WFA (initially resuspended in Dimethyl Sulfoxide and then diluted with Glycerol Trioctanoate) once every 3 days at 2 mg/kg or 6 mg/kg. The lower dosage was selected as it has been found to be a therapeutic dosage in multiple studies and the upper dosage was selected to test the effects of a supratherapeutic dosage. Our desired endpoint was 5 weeks post-tumor implantation. However, the mice were euthanized 29 days post-implantation due to several mice reaching the criteria for an early humane endpoint. Post-euthanization, several tissues were collected, weighed, snap frozen in liquid nitrogen and then stored at − 80 °C for further analysis or fixed in buffered formalin solution. While alive, the mice were housed in a 12 h light–dark cycle and given water and food ad libitum. The Institutional Animal Care and Use Committee (IACUC, protocol # 15405) and Institutional Biosafety Committee (IBC, protocol # 18–208) of the University of Louisville approved all experimental protocols in mice in advance.

### Grip strength measurements

Protocol used for the measurement of grip strength was essentially similar to as described previously [28]. Before assessment, mice were weighed on a commercially available digital scale. Forepaw and total grip strength of mice were measured using a digital grip strength meter (Columbus Instruments, Columbus, OH, USA) and then normalized by total body weight. Before the beginning of the test, the mice were acclimatized for 5 min. The mouse was allowed to grasp the total paw pull-bar assembly, and in a separate experiment the forepaw pull-bar assembly. The mouse was then gently drawn with constant force in a straight line away from the device until the mouse could no longer grasp the bar. Force at time of release was recorded as the peak tension. Each mouse was tested five times with a delay of 20–40 s between each testing. The mean peak tension was calculated from the recordings normalized by total body weight.

### Analysis of body composition

Body fat and lean mass composition, as well as the bone mineral content and density, excluding head region of interest (ROI), were determined by dual-energy X-ray absorptiometry (DEXA) scan using a mouse densitometer (PIXImus2; Lunar, Madison, WI, USA) as previously described [29]. According to the manufacturer’s guidelines to calibrate and to validate the performance of the apparatus, a “mouse phantom” provided with the machine was scanned before scanning the first mouse. Animals were euthanized immediately before assessment by DEXA scan. The mouse was then transferred to the disposable PIXImus measuring tray in the prone position with their head in a loosely fitting nose cone to aid positioning. Upon completion of the DEXA scan, approximately 5 min, the mouse was placed on ice to preserve tissue prior to collection. Quality control report is available upon request.

### Histology and morphometric analysis of skeletal muscle

The tibialis anterior (TA), gastrocnemius (GA) and quadriceps femoris (QF) of the mice were isolated, flash frozen in liquid nitrogen, mounted in O.C.T. embedding medium, and then sectioned using a microtome cryostat. To assess tissue morphology, 10 μm thick transverse sections were cut from the mid-belly of the muscle and then subjected to Hematoxylin and Eosin (H&E) staining. Images of H&E-stained TA/GA/QF muscle sections were quantified using Fiji software (National Institute of Health software) to measure myofiber cross-sectional area (CSA). Myofiber CSA was calculated by analyzing ~ 350–500 myofibers per muscle as previously described [30].

### Total RNA purification and qPCR

Isolation of total RNA from tumor samples was performed using an RNeasy Mini Kit (Qiagen Catalog # 74104) according to the manufacturer’s instructions. Isolation of total RNA from skeletal muscle was performed using an RNeasy Fibrous Tissue Mini Kit (Qiagen Catalog # 74704) according to the manufacturer’s instructions. First strand cDNA was synthesized using 1 μg of purified RNA and a commercially available kit (iScript™ cDNA synthesis, Bio-Rad Catalog # 170–8891), Quantification of mRNA expression was performed using real-time PCR as described previously [31] using the SYBR Green dye method on a StepOnePlus™ system (Applied Biosystems) and gene-specific primers as detailed in Additional file 1: Table S1.

### Skeletal muscle fiber-type immunostaining

Similar to as described [28], to determine the composition of different types and gross composition of skeletal muscle fibers, 10 μm thick transverse sections were made from TA muscles, a hydrophobic boundary was drawn around the section, and then blocked with 5% goat serum and 2% bovine serum albumin (BSA) in PBS for 30 min. The sections were then incubated for 1 h with monoclonal antibodies against Myosin Heavy Chain (MyHC) isoforms type I, IIa, and IIb using clone BA-D5, SC-7, and BF-F3, respectively (Developmental Studies Hybridoma Bank, Iowa City, IA, USA). The following secondary antibodies were then used for detection after 1–2 h of incubation period: anti-Mouse IgG2b (γ2b) CF™350 antibody produced in goat, anti-Mouse IgG1 (γ1) CF™568 antibody produced in goat, and Alexa Fluor-488 goat anti-mouse IgM. Fluorescence images were captured with a Nikon TiE 3000 inverted microscope, the single color channels were merged, and percentage of each fiber-type in whole muscle section was recorded. We determined nonspecific background staining by performing the above protocol, but omitting the inclusion of the primary antibodies. Antibodies BA-D5, SC-71, and BF-F3 were deposited to the DSHB by Schiaffino, S. (DSHB Hybridoma Product BA-D5, SC-71, and BF-F3). Refer to Additional file 2: Table S2 for a complete list of antibodies utilized.

### Immunofluorescent immunohistochemistry of tumor tissues

For the detection of various proteins in tumor tissues, 10 μm thick sections were made from the midsection of tumor samples. A hydrophobic boundary was drawn around the sections and then were fixed in 3.7% formaldehyde solution for 10 min. The slides were washed three times with 1X PBS for 5 min each. The sections were then blocked with 2% BSA in PBS for 30 min. The sections were then incubated with primary antibodies (1:100 dilution) overnight in a humidified chamber at 4 °C followed by three washes with 1X PBS for 5 min each. The slides were incubated in appropriate secondary antibodies (1:2000 dilution) for 45–60 min, and were counterstained with DAPI (1:2500 dilution) for 5 min to visualize nuclei. Slides were washed twice in 1X PBS for 5 min, and then mounted for visualization. Nonspecific background staining was determined by performing the above protocol with omitting the inclusion of the primary antibodies. Refer to Additional file 2: Table S2 for a complete list of antibodies utilized.

### Imaging

Slides were mounted using Eukitt Quick-hardening mounting medium (Sigma-Aldrich) and visualized at − 0.4 °C on a Nikon TiE 3000 inverted microscope (Nikon) equipped with a digital camera (DS-U2/L2-Ri1 digital microscope camera (Nikon) for light microscopy or DXM-1200C coded digital camera (Nikon) for fluorescent microscopy), and Nikon NIS Elements AR software (Nikon). Exposure times were consistent for each staining type. Image levels were equally adjusted using Adobe Photoshop CS6 software (Adobe) to remove nonspecific background staining.

### Human MAP multiplex assay

Milliplex MAP Human Il-18 Singleplex Magnetic Bead Kit (Millipore Sigma Catalog # HIL-18MAG-55 K, Lot # 3179880) was reconstituted as per the manufacturer’s protocol and utilized to reconstitute the Milliplex MAP Human Cytokine/Chemokine Magnetic Bead Panel (Millipore Sigma Catalog # HCYTOMAG-60 K, Lot # 3203050, 9 Analytes) according to the manufacturer’s instructions. Reconstituted MAP multiplex was performed according to manufacturer’s protocols with the exception of the following modifications. MAP Cytokine Assay Buffer LE-ABGLP2 (Millipore Sigma Catalog # LE-ABGLP2) was used instead of the one provided with the kit to lyse tumor samples/extract protein at the recommendation of the manufacturer. Briefly, tumor samples were bisected over dry ice and then transferred into a 1.5 ml micro-centrifuge tube containing 250 μl MAP Cytokine Assay Buffer per 100 mg tissue. Samples were minced on ice with sterilized scissors. Minced tissue was subjected to sonication for 30 s on ice. Gross cellular debris was removed by centrifugation at 10,000 RPM for 3 min at room temperature. Four blanks were employed instead of two and an additional dilution was included in the standard curve (0.64 pg/ml). Two concentrations of the samples (10 and 50 μg total protein) were applied in triplicate. MAP Cytokine Assay Buffer was used for the blank wells and to dilute samples.

### Graphical display and statistical analyses

The majority of the results were expressed as box-and-whisker plots with the box comprised of the first, second, and third quartiles, and the lower and upper whiskers corresponding to the minimum and maximum values, respectively, to display the entire range of data. Individual data points are depicted as opaque black circles. A Kaplan-Meier curve was used for the survival analysis. Statistical analysis of the data was performed using one-way analysis of variance (ANOVA) followed by Tukey’s Honestly Significant Difference Test (HSDT) post hoc analysis or the Mantel-Cox log-rank test to determine statistically significant differences between groups with GraphPad Prism 8.0.1 software for Mac (La Jolla, California, USA). A value of *P* < 0.05 was considered statistically significant, unless otherwise specified.

## Results

### Withaferin A impedes the increase in mortality and body composition changes associated with ovarian cancer

**Fig. 1:**
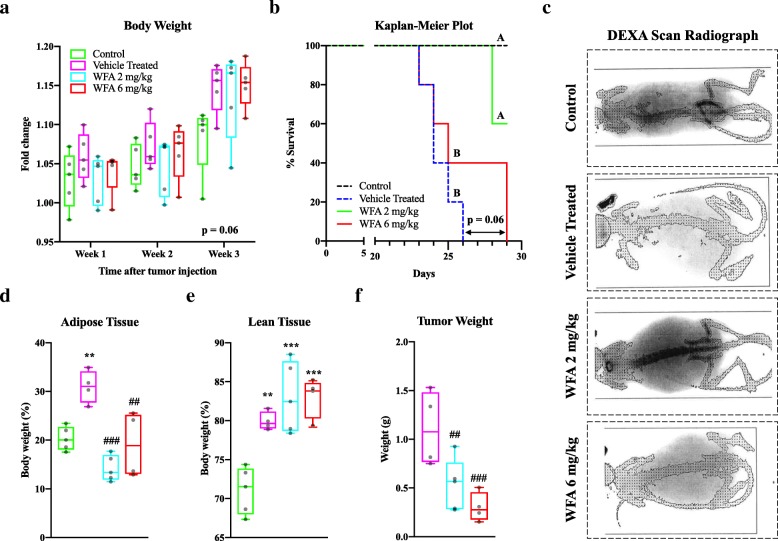
Withaferin A decreases the mortality rate and body composition changes associated with ovarian cancer. (**a**) Body weight fold change of control, vehicle treated, WFA 2 mg/kg, and WFA 6 mg/kg groups over the period of the study. *N* = 5 in each group. (**b**) Kaplan-Meier survival analysis. Letters A and B are used to delineate groups with respect to statistical significance (A = control and WFA 2 mg/kg; B = WFA 4 mg/kg and vehicle treated). (**c**) Representative DEXA scan images of control, vehicle treated, WFA 2 mg/kg, and WFA 6 mg/kg mice. Area within dashed box surrounding image is the same. Quantitative estimation of whole body DEXA scan minus head ROI of (**d**) adipose tissue and (**e**) lean tissue as a percentage of body weight. (**f**) Average weight of visible tumors in the peritoneum. N = 5 (control), 4 (vehicle treated), 5 (WFA 2 mg/kg), and 4 (WFA 6 mg/kg). Opaque black circles indicate individual data points. *P < 0.05; **p < 0.01; ***p < 0.001, value significantly different from corresponding value of control group as determined by one-way ANOVA followed by Tukey’s HSDT. ^**#**^*P* < 0.05, value significantly different from corresponding value of vehicle treated group. ^**$**^P < 0.05, value significantly different from corresponding value of WFA 2 mg/kg group

To assess the effects of WFA on ovarian cancer-induced cachexia, female severely immunodeficient NSG mice received intraperitoneal (i.p.) injections of A2780 cells to generate a xenograft model of ovarian cancer or sterile saline to assess the normal development of mice, henceforth referred to as control group. After an initial lag phase, tumor-bearing mice were treated with one of the two doses of WFA (2 mg/kg or 6 mg/kg) or vehicle, henceforth referred to as vehicle treated group. The control group also received injections of vehicle. We recorded a baseline body weight from all mice 1 day after i.p. injection of A2780 cells, and subsequently every week until the end of the study (Table 1). No statistically significant difference between the groups, as determined by one-way analysis of variance (ANOVA), was found by the termination of the study in body weight fold change (Fig. 1a). However, there was a trend towards an increase (F(3, 16) = 2.874 with an associated *p*-value of 0.06) in change in body weight of tumor bearing groups at the endpoint, which is due to an increase in tumor mass. The study was intended to last for 5 weeks post-tumor implantation, however, shortly after our assessment at week 3, mice had begun to succumb to tumor burden or met the criteria for an early humane endpoint. A survival analysis was performed amongst the four groups to elucidate whether treatment had any effect on mortality (Fig. 1b). To analyze the survival Kaplan-Meier plot, the Mantel-Cox log-rank test was performed and an associated *p*-value of 0.0003 was determined (χ^2^ = 19.17, df = 3). We found no significant difference between the control group and the group of mice treated with 2 mg/kg of WFA, but these groups showed a higher survival rate compared to WFA 6 mg/kg and vehicle treated groups (Fig. 1b). No significant difference between the 6 mg/kg WFA and vehicle treated groups was found, although there was a trend towards an increase in survival (*p* = 0.06) in the WFA 6 mg/kg group. We assessed the normalized weight of selected vital organs due to the differences in survival (Table 1). Despite evidence of several tumors found throughout the peritoneal cavity, we found no statistically significant changes in our assessment of selected vital organs.

**Table 1:** Quantitative assessment of body weight and select organ weight

Organ	Control	Vehicle Treated	WFA (2 mg/kg)	WFA (6 mg/kg)
BW (Initial) (g)	18.71 ± 0.91	18.96 ± 2.11	19.14 ± 1.50	19.60 ± 0.76
BW (final) (g)	20.83 ± 1.53	22.65 ± 2.00	22.68 ± 2.53	23.57 ± 1.23
Kidney (mg/g)	6.26 ± 0.57	6.08 ± 1.03	4.95 ± 0.79	5.45 ± 0.49
Liver (mg/g)	53.67 ± 4.65	44.54 ± 3.64	47.16 ± 15.88	41.18 ± 3.60
Spleen (mg/g)	1.41 ± 0.53	2.06 ± 0.11	2.20 ± 1.74	2.78 ± 1.45
Heart (mg/g)	5.56 ± 1.12	4.20 ± 0.21	4.97 ± 1.96	3.74 ± 0.23

Gross body weight was recorded for each group over the course of the study. Selected organ weights were recorded and then normalized to final body weight of corresponding mice

Data displayed as x̅ ± σ. BW = Body weight

In addition to an increased rate of mortality, cachexia generally results in the wasting of skeletal muscle [9]. In order to determine whether ovarian cancer or WFA had any effect on body composition, we assessed the carcasses by dual-energy X-ray absorptiometry (DEXA) scan. In Fig. 1c, we show representative DEXA scan images from control, vehicle treated, WFA 2 mg/kg, and WFA 6 mg/kg groups. As determined by one-way ANOVA and Tukey’s Honestly Significant Difference Test (HSDT), we found a statistically significant increase in the proportion of adipose tissue to whole body weight in the vehicle treated group compared to the control group (F(3, 14) = 13.67; *p* = 0.006) (Fig. 1d), consistent with published reports linking a generalized accumulation of adipose tissue in the thoracoabdominal region with ovarian cancer [32]. We found an attenuation of adipose tissue following treatment of WFA at both concentrations compared to the vehicle treated group (WFA 2 mg/kg: *p* = 0.0001; WFA 6 mg/kg: 0.0040) (Fig. 1d). Paradoxically, we observed a significant increase in the proportion of lean tissue to body weight in all tumor-bearing groups regardless of treatment with WFA (F(3, 14) = 15.13; vehicle treated: *p* = 0.0048; WFA 2 mg/kg: *p* = 0.0002; WFA 6 mg/kg: *p* = 0.0004), contrary to what one would expect in a cachectic state (Fig. 1e) [9]. Upon examination of the DEXA scan images, it appears that the solid tumors might have the same radiographic signature as the lean tissue, potentially explaining the paradoxical increase in lean tissue. No significant differences were found in bone mineral density or content (Data Not Shown).

Free peritoneal tumors (visible tumors) not associated with any of the organ were collected and weighed to assess WFA effects (Fig. 1f). We found that WFA significantly reduced the size of peritoneal tumors as compared to the vehicle treated group (F(2, 10) = 8.746; WFA 2 mg/kg: *p* = 0.03; WFA 6 mg/kg: *p* = 0.0061). No significant difference was observed between the two doses of WFA (*p* = 0.48) (Fig. 1f), suggesting that WFA inhibits tumor growth in NSG mice. At the time of euthanization and tissue collection, we noted that tumors associated with the intestines and ovaries were found most frequently in the vehicle treated group and were of the largest size (Data Not Shown**).** In contrast, WFA treatment groups resulted in fewer and smaller tumors, with the 6 mg/kg group having the smallest tumors (Data Not Shown) in ovaries, indicating reduction in tumor metastasis. Cumulatively, our data indicates that treatment with WFA improves the survival of mice xenografted with A2780 cells and rescues some of the body changes associated with ovarian cancer. Our DEXA scan data in conjunction with the collection and assessment of intraperitoneal tumors suggests that there might be attenuation in the loss of lean tissue, and therefore skeletal muscle, but we cannot conclusively determine this due to the radiographic abnormalities exhibited by the xenografted tumors.

### Withaferin A treatment partially rescues the gonadal fat pad

Ovarian cancer progression is often associated with the specific loss of the gonadal fat pad [11, 32]. As such, we attempted to quantify the amount of adipose tissue present in the gonadal region at the time of tissue collection post-euthanization. The severe reduction in the gonadal fat pad rendered accurate measurement of gonadal adipose tissue improbable. Further, in some mice, there was a complete absence of gonadal fat. As such, qualitative remarks about the gonadal fat pad are mentioned below. In the tumor-free control group, mice exhibited an abundance of adipose tissue surrounding in the gonadal region. Consistent with published reports linking specific changes in the gonadal fat pad to the progression of ovarian cancer, all mice within the vehicle treated group had a complete absence of fat in the gonadal region [11, 32]. The mice treated with WFA had scant amounts of adipose tissue, but not enough in such a quantity that could be accurately weighed. While admittedly qualitative, this data is indicative that treatment with WFA impedes, but does not completely resolve body composition changes caused by ovarian cancer.

### Withaferin A treatment functionally rescues muscle strength in ovarian cancer-induced cachexia

**Fig. 2:**
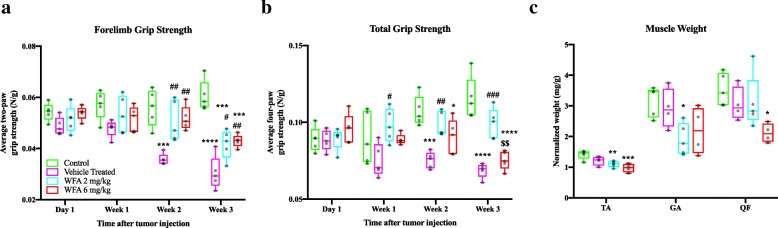
Withaferin A ameliorates muscle strength in ovarian cancer-induced cachexia. Quantification of mean (**a**) forelimb and (**b**) total limb grip strength normalized to body weight at time of assessment. *N* = 5 in all groups. (**c**) Quantification of normalized tibialis anterior (TA), gastrocnemius (GA), and quadriceps femoris (QF) muscle weights to total final body weight. *N* = 5 (control), 4 (vehicle treated), 5 (WFA 2 mg/kg), and 4 (WFA 6 mg/kg). Opaque black circles indicate individual data points. *P < 0.05; **p < 0.01; ***p < 0.001; ****p < 0.0001, value significantly different from corresponding value of control mice as determined by one-way ANOVA followed by Tukey’s HSDT. ^**#**^*P* < 0.05, value significantly different from corresponding value of vehicle treated group. ^**$**^*P* < 0.05, value significantly different from corresponding value of WFA 2 mg/kg group

A major hallmark of cachexia is a reduction in muscle strength [9]. Therefore, we examined whether ovarian cancer or WFA had an effect on forelimb grip strength. We observed a steady reduction in normalized forelimb grip strength in the vehicle treated group compared to the control group, reaching statistical significance at week 2 post-xenografting of A2780 cells (F(3, 16) = 11.01 (week 2); *p* = 0.0003) (Fig. 2a). The mice treated with WFA showed a reduction in normalized grip strength compared to the control group, reaching statistical significance at week 3 post-implantation (F(3, 16) = 26.64 (week 3); WFA 2 mg/kg: p = 0.0003; WFA 6 mg/kg: *p* = 0.0005) (Fig. 2a). Despite the reduction, there was an attenuation of normalized forelimb grip strength following treatment of WFA compared to the vehicle treated group, achieving statistical significance by week 2 post-injection of A2780 cells (WFA 2 mg/kg: *p* = 0.0058; WFA 6 mg/kg: *p* = 0.0029) (Fig. 2a). No statistical difference between both doses of WFA was found (*p* = 0.9861) (Fig. 2a). In a separate experiment where we measured the normalized 4-paw (total limb) grip strength, the vehicle treated group showed a profound reduction in normalized total limb grip strength compared to the control group, achieving statistical significance by week 2 of post-injection of A2780 cells (F(3, 16) = 11.61 (week 2); *p* = 0.0002) (Fig. 2b). Treatment with 2 mg/kg of WFA showed a significant protective effect on total limb grip strength throughout the period of the study compared to the vehicle treated group (F(3, 16) = 3.480 (week 1), 11.61 (week 2), and 26.62 (week 3); *p* = 0.03, 0.0039, and 0.005, respectively) (Fig. 2b). Treatment with 6 mg/kg of WFA was initially protective, but the grip strength eventually deteriorated to the same extent as the vehicle treated group (Fig. 2b), which could be due to the development of toxicity by this high dose of WFA. Based on the increased mortality rate of the WFA 6 mg/kg and the decline in grip strength similar to that of the vehicle treated group, we believe that 6 mg/kg of WFA is a lethal dose for these animals.

In conjunction with a loss of muscle strength, a reduction in skeletal muscle mass is characteristic of cachexia, including cancer-induced cachexia [9, 10]. Therefore, we investigated whether there were changes in the normalized weight of select muscles of the lower extremity (Fig. 2c). We found a significant reduction in normalized wet weight of the tibialis anterior (TA) of all tumor-bearing mice treated with WFA compared to the control group (F(3, 14) = 8.876; WFA 2 mg/kg: *p* = 0.01; WFA 6 mg/kg: *p* = 0.001) (Fig. 2c). The gastrocnemius (GA) of the WFA 2 mg/kg group showed a significant reduction in normalized wet weight compared to the control group (F(3, 14) = 4.591; *p* = 0.02) (Fig. 2c). The quadriceps femoris (QF) of the WFA 6 mg/kg group displayed a significant reduction in wet weight compared to the control group (F(3, 14) = 3.968; *p* = 0.02) (Fig. 2c). Curiously, no difference between the vehicle and WFA treated groups was found.

**Fig. 3:**
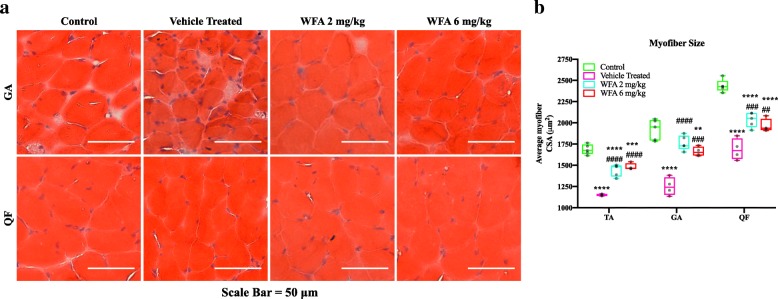
Withaferin A ameliorates myofiber size reduction in ovarian cancer-induced cachexia. (**a**) Representative images of H&E stained GA and QF transverse muscle sections. (**b**) Quantification of average myofiber cross-sectional area (CSA). Scale Bar = 50 μm. *N* = 5 (control), 4 (vehicle treated), 5 (WFA 2 mg/kg), and 4 (WFA 6 mg/kg). Opaque black circles indicate individual data points. *P < 0.05; **p < 0.01; ***p < 0.001; ****p < 0.0001, value significantly different from corresponding value of control mice as determined by one-way ANOVA followed by Tukey’s HSDT. #*P* < 0.05, value significantly different from corresponding value of vehicle treated group. $*P* < 0.05, value significantly different from corresponding value of WFA 2 mg/kg group

To investigate the discrepancy between a reduction in grip strength and no changes in muscle weight between the vehicle and WFA treated groups, we performed Hematoxylin and Eosin (H&E) staining on transverse sections of the TA, GA, and QF muscles (Fig. 3a, TA images not shown) and measured the myofiber cross-sectional area (CSA) (Fig. 3b). In all three skeletal muscles, there was a significant reduction in average myofiber CSA in the vehicle treated group compared to the control group (F(3, 14) = 75.62 (TA), 37.58 (GA) and 51.29 (QF); *p* < 0.0001 for all muscles) (Fig. 3b). Both doses of WFA resulted in CSAs that were significantly greater than the vehicle treated group for all three muscles examined (WFA 2 mg/kg: *p* < 0.0001 (TA & GA), *p* = 0.0003 (QF); WFA 6 mg/kg: *p* < 0.0001 (TA), *p* = 0.0002 (GA), *p* = 0.004 (QF) (Fig. 3b). In the TA and QF muscles, there was a significant reduction in average myofiber CSA in the WFA treated groups compared to the control group (WFA 2 mg/kg: *p* < 0.0001 (TA & QF); WFA 6 mg/kg: *p* = 0.0003 (TA), *p* < 0.0001 (QF)) (Fig. 3b). We found a restoration of myofibrillar CSA in the GA muscle of mice treated with 2 mg/kg of WFA (*p* = 0.09), but not those treated with 6 mg/kg of WFA (*p* = 0.007) compared to the control group (Fig. 3b). While a reduction in average CSA was found, there was only a weak trend towards an increase in the number of myofibers present in the tumor bearing groups that did not approach statistical significance (Data Not Shown). A scant degree of skeletal muscle edema was present in select muscles of tumor-bearing mice, but not to an extent that would mask weight changes (Data Now Shown). While a definite answer was not elucidated for the grip strength/muscle weight discrepancy, we found that treatment with WFA partially rescues grip strength and myofibrillar CSA in our cachectic model, demonstrating a functional improvement in our xenografted cancer model.

### WFA abolishes the slow-to-fast myofiber-type conversion associated with cancer cachexia

**Fig. 4:**
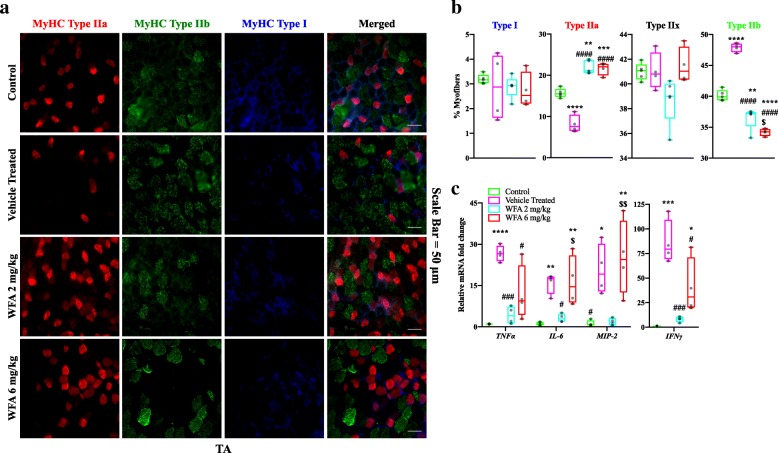
Withaferin A inhibits a slow to fast fiber-type conversion in ovarian cancer. (**a**) Representative images of transverse TA muscle sections from control, vehicle treated, WFA 2 mg/kg, and WFA 6 mg/kg mice subjected to triple immunostaining against MyHC I (blue color), IIa (red color), and IIb (green color) proteins. Unstained fibers were considered to be Type IIx. Scale Bar = 50 μm. (**b**) Quantification of the percentage of Type I, Type IIa, Type IIx, and Type IIb myofibers in TA muscle. (**c**) Relative mRNA levels of select inflammatory cytokines in TA muscle. *N* = 4 in each group. Opaque black circles indicate individual data points. *P < 0.05; **p < 0.01; ***p < 0.001; ****p < 0.0001, value significantly different from corresponding value of control group as determined by one-way ANOVA followed by Tukey’s HSDT. ^**#**^*P* < 0.05, value significantly different from corresponding value of vehicle treated group. ^**$**^*P* < 0.05, value significantly different from corresponding value of WFA 2 mg/kg group

Skeletal muscle wasting in response to various pathological states, including cancer-induced cachexia, is associated with the conversion of slow skeletal muscle fibers (Type IIa) to fast skeletal muscle fibers (Type IIb) in certain muscles, such as the soleus and TA [33]. Further, studies have shown that fast skeletal muscle fibers exhibit an accelerated rate of atrophy [34, 35]. To assess myofiber composition changes, we performed immunohistochemistry (IHC) to detect expression of selected isoforms of Myosin Heavy Chain (Fig. 4a). Unstained myofibers were considered to be type IIx, which are generally considered to be myofibers that are transitioning fiber-type [36]. Consistent with published reports [37], Type I and IIa fibers were found clustered towards the inner aspect of the TA muscle and the myofiber distribution became predominantly Type IIb and Type IIx as you moved laterally from the centralized clusters (Fig. 4a). Also consistent with published reports [37], Type I myofibers made up a small amount of the myofibers in the TA muscle in all groups, and no significant differences were found (F(3, 14) = 0.2942 with an associated *p*-value of 0.83) (Fig. 4b). We found a significant reduction in the percentage of Type IIa myofibers in the vehicle treated group compared to all other groups (F(3, 14) = 67.78; *p* < 0.0001 for all comparisons) (Fig. 4b). Interestingly, we found a significant increase in Type IIa myofibers in both groups treated with WFA compared to the vehicle treated and control groups (WFA 2 mg/kg: *p* = 0.0001; WFA 6 mg/kg: *p* = 0.0004) (Fig. 4b). For the Type IIx myofibers, one-way ANOVA indicated a significant difference (F(3, 14) = 3.603 with an associated p-value of 0.04) (Fig. 4b). However, no significant differences were elucidated upon post hoc analysis (Fig. 4b). In contrast, we found a significant increase in the proportion of Type IIb myofibers in the vehicle treated group compared to all groups (F(3, 14) = 110.5; *p* < 0.0001 for all comparisons) (Fig. 4b). Further, the amount of Type IIb myofibers was significantly reduced in the WFA treated groups in a dose-dependent manner, as compared to the control (WFA 2 mg/kg: *p* = 0.001; WFA 6 mg/kg: *p* < 0.0001) and vehicle treated groups (p < 0.0001 for both groups) (Fig. 4b). Taken together, our experiments clearly demonstrate that ovarian cancer induces skeletal muscle cachexia, which is ameliorated by WFA. Interestingly, we note that treatment with WFA resulted in more Type IIa and fewer Type IIb myofibers than what was exhibited in the control group.

### WFA impedes transcriptional changes associated with myofiber-type conversion

Studies have shown that systemic expression of tumor secreted proinflammatory cytokines downstream of the NF-κB pathway is capable of orchestrating myofiber atrophy and fiber-type conversion [38]. Therefore, we investigated classical proinflammatory cytokines downstream of the NF-κB pathway (Fig. 4c). Relative mRNA levels of all proinflammatory cytokines assessed (*TNFα*, *IFNγ*, *IL-6*, and *MIP-2*) were significantly increased in the TA muscle of vehicle treated mice compared to the control group (F(3, 12) = 17.41, 18.25, 10.56, and 9.22, respectively; *p* = 0.0001 (*TNFα* and *IFNγ*), *p* = 0.0062 (*IL-6*), and *p* = 0.03 (*MIP-2*)) (Fig. 4c). Transcript levels of these inflammatory cytokines were significantly reduced in the WFA 2 mg/kg group compared to the vehicle treated group (*p* = 0.004 (*TNFα*), *p* = 0.003 (*IFNγ*), *p* = 0.02 (*IL-6*), and p = 0.03 (*MIP-2*), and were not significantly different than the control group (*p* = 0.84, 0.95, 0.94, and 0.99, respectively) (Fig. 4c). Curiously, we observed a robust increase in the expression of selected proinflammatory cytokines in the WFA 6 mg/kg group compared to the control (*p* = 0.04 (*IFNγ*), 0.005 (*IL-6*), 0.007 (*MIP-2*), and 0.06 (*TNFα*)) and WFA 2 mg/kg groups with respect to *IL-6* and *MIP-2* expression (*p* = 0.02 and 0.008, respectively) (Fig. 4c), likely as a byproduct of the toxicity of the high dosage. Relative transcript levels of *IFNγ* and *TNFα* were significantly reduced in the WFA 6 mg/kg group compared to the vehicle treated group (*p* = 0.02 for both genes) (Fig. 4c). Collectively, our results indicate that low doses of WFA systemically downregulate classical downstream targets of the NF-κB pathway at a transcriptional level, which are known to mediate myofibrillar atrophy and myofiber-type conversion.

### WFA inhibits the production of NF-κB-related proinflammatory cytokines in a xenografted model of ovarian cancer

**Fig. 5:**
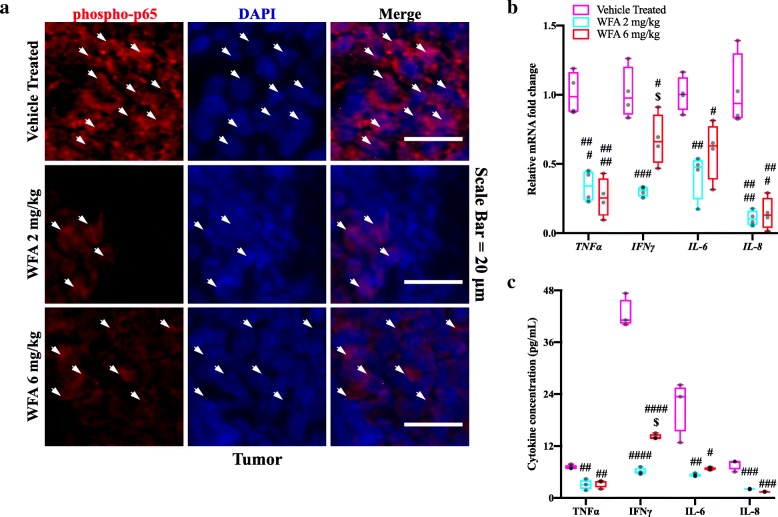
Withaferin A impedes activation of NF-κ-related inflammatory cytokines. (**a**) Representative images of tumor samples subjected to immunofluorescent immunohistochemical staining against phospho-p65 (red color). Nuclei were visualized with DAPI counterstaining (blue color). Arrow points to phospho-p65^+^ nuclei. Scale Bar = 20 μm. *N* = 4, 5, 4, respectively. (**b**) Relative mRNA levels of select inflammatory cytokines in tumor samples. *N* = 4 in each group. (**c**) Concentration of select cytokines (pg/ml) in tumor samples. *N* = 4 in each group. Opaque black circles indicate individual data points. ^**#**^*P* < 0.05; ^**##**^*p* < 0.01; ^**###**^*p* < 0.001; ^**####**^*p* < 0.0001, value significantly different from corresponding value of vehicle treated group as determined by one-way ANOVA followed by Tukey’s HSDT. ^**$**^*P* < 0.05, value significantly different from corresponding value of WFA 2 mg/kg group

In ovarian cancer, proinflammatory cytokines are linked to NF-κB pathway and are often upregulated [39]. WFA has been reported to regulate the NF-κB pathway through inhibiting IKKβ activation [40] and NF-κB-DNA binding [41]. Further, it has been shown to decrease levels of phospho- and total-p65, the active canonical NF-κB transcription factor [41]. Therefore, we investigated whether various proinflammatory cytokines are being regulated in response to WFA treatment in the xenografted tumor (Fig. 5). In our study, we found that tumor samples from the vehicle treated group showed the highest levels of phospho-p65, the active form of the canonical NF-κB signaling protein, (Fig. 5a). We assessed tumor samples for the expression of phospho-p65 using immunofluorescent IHC (Fig. 5a). Phospho-p65 was found in nearly all of the tumor cells in the vehicle treated group (Fig. 5a). A striking reduction in cells positive for phospho-p65 was readily observed upon treatment with WFA (Fig. 5a). In addition to a reduction in phospho-p65 staining, we also observed a reduced amount of phospho-p65 co-localized with DAPI, indicating impairment in the translocation of p65 to the nucleus (Fig. 5a). Next, we assessed the expression of select NF-κB related proinflammatory cytokines in the xenografted tumor samples (Fig. 5b). Similar to the results in Fig. 4c, relative transcript levels of all proinflammatory cytokines assessed (*TNFα*, *IFNγ*, *IL-6*, and *IL-8* (the human ortholog of mouse *MIP-2*) were significantly reduced in the WFA 2 mg/kg treated group compared to the vehicle treated group (F(2,9) = 36.05, 21.93, 12.71, and 38.37, respectively; *p* = 0.0002, 0.0003, 0.0021, and < 0.0001, respectively) (Fig. 5b). Relative levels of mRNA expression of the aforementioned proinflammatory cytokines were significantly reduced in the WFA 6 mg/kg treated group compared to the vehicle treated group (*p* < 0.0001, = 0.03, 0.02, and 0.0001, respectively) (Fig. 5b). Relative mRNA levels of *IFNγ* were significantly increased in the WFA 6 mg/kg group compared to the WFA 2 mg/kg group (*p* = 0.02). No significant differences were observed in the other genes (*p* = 0.68 (*TNFα*), 0.33 (*IL-6*), and 0.96 (*IL-8*) (Fig. 5b). As a final level of confirmation, we performed a human MAP multiplex assay to assess protein levels of selected proinflammatory cytokines related to the NF-κB pathway (TNFα, IFNγ, IL-6, and IL-8; F(2, 6) = 15.59, 201.4, 13.12, and 54.46, respectively) (Fig. 5c). We found that treatment with a low dose of WFA significantly reduced the expression of all proinflammatory cytokines tested compared to the vehicle treated group (*p* = 0.006, < 0.0001, 0.008, and 0.0004, respectively) (Fig. 5c). The high dose of WFA resulted in a downregulation of proinflammatory cytokines compared to the vehicle treated group (*p* = 0.008, < 0.0001, 0.01, and 0.0002, respectively) (Fig. 5c). Levels of IFNγ were increased in the WFA 6 mg/kg group compared to the WFA 2 mg/kg group (*p* = 0.02) (Fig. 5c), which could be due to the development of toxicity by this high dose of WFA. No other significant differences were observed between these two groups. Collectively, our results demonstrate that WFA modulates activation of the canonical NF-κB pathway and downstream transcriptional activities, indicating the mechanism through which WFA ameliorates cachectic signaling in our xenografted ovarian cancer model.

## Discussion

Cancer cachexia has been recognized as a severe byproduct of numerous cancers for several years, and negatively affects the survival outcome and quality of life of cancer patients [42]. A recent report analyzed the prevalence of cachexia in 14 different cancer types known to exhibit cachexia or a muscle wasting syndrome within the United States and the European Union, and found that over 1.3 million cancer patients exhibited clinical signs of cachexia in these oncological settings [42]. Reports have shown that cachexia is mediated, at least in part, by canonical NF-κB signaling [39, 43]. As such, a thoughtful examination of this signaling paradigm could pave the way for novel therapeutics to address this current deficit.

Based on current literature, it appears that ovarian cancer could be an ideal model to understand and target cachexia due to its upregulation of NF-κB signaling and high incidence of cachexia. A recent report established a xenograft model of ovarian cancer in mice, where the researchers’ mimicked the clinical symptoms of cachexia in ovarian cancer patients [11]. Indeed, they report that xenografting of the ovarian cancer cell line ES-2 resulted in a profound cachectic phenotype, exhibiting gross body changes and functional muscle weakening, consistent with cachexia symptoms in humans [11]. In our study, we generated an ovarian cancer xenograft i.p. model and then attempted to reverse the cancer-induced cachectic phenotype. We were able to recapitulate the essence of the aforementioned report, but also evidence an amelioration of the cachectic state. Consistent with clinical symptoms of ovarian cancer and cachexia [7, 8], our vehicle treated mice showed gross body changes, a reduction in survival, and functional muscle decline due to tumor burden. Promisingly, treatment with WFA significantly rescued many of these parameters (Figs. 1 and 2).

Withaferin A is known to inhibit the activation of the canonical NF-κB pathway at defined steps, specifically activation of the IKK complex and translocation of p65 to the nucleus [40, 41]. Therefore, we refined our investigation to signaling paradigms immediately distal of the IKK complex (Figs. 4 and 5). A key consequence of sustained activation of the canonical NF-κB pathway is the ubiquitous production and release of proinflammatory cytokines, such as TNFα and IL-6 [44]. During the acute phase of muscle injury, activation of the NF-κB pathway and subsequent production of proinflammatory is initially beneficial to the injured microenvironment and facilitates the initial phases of the repair process [45]. However, prolonged exposure to these proinflammatory cytokines/chemokines rapidly becomes detrimental to skeletal muscle [45]. Cancers are notorious for their ubiquitous expression of proinflammatory cytokines, ovarian cancer being no exception [44]. However, WFA acts systemically so the possibility of multiple cell crosstalk should not be dismissed. In the current study, we refined out focus to the xenografted tumors and resident skeletal muscle without analysis of other tissues, such as hematopoietic stem cells that are known to facilitate in regenerative myogenesis [46].

**Fig. 6:**
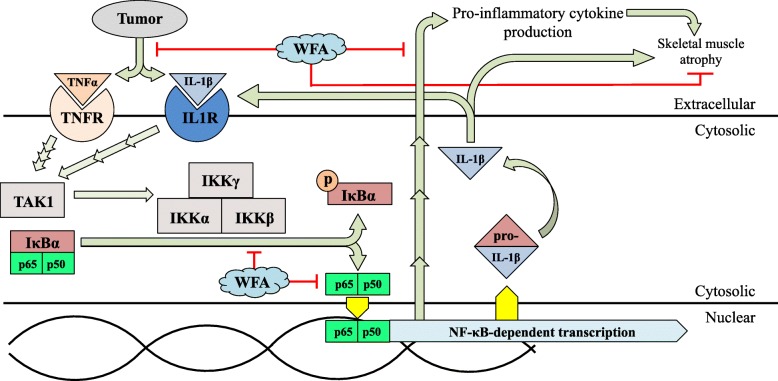
Proposed mechanisms of Withaferin A effect on ovarian cancer-induced cachexia. Mechanistic model showing WFA effect on upstream cachectic signaling in ovarian cancer

Due to the anti-inflammatory properties of WFA and its direct inhibition of NF-κB signaling, we hypothesized that WFA could ameliorate the cachectic phenotype exhibited in ovarian cancer. Indeed, we report molecular changes in the canonical NF-κB signaling pathway and the downstream consequences of this modulation. Figure 6 illustrates the proposed mechanistic action of WFA and how it inhibits ovarian cancer-induced cachexia via a direct regulation of canonical NF-κB signaling and downstream effects blunting production of proinflammatory cytokines known to induce atrophic changes. Consistent with published reports [44], we found that the vehicle treated group had a high expression of select proinflammatory cytokines (Figs. 4 and 5). Treatment with WFA led to a robust decrease in the levels of these proinflammatory cytokines and reduced their transcriptional activation in the tumors, as well as in resident skeletal muscle. As a byproduct of this reduced inflammatory environment, the skeletal muscle myofibers were not atrophied to the extent as the vehicle treated group, displaying a promising effect of WFA.

However, tumors are complex environments and do not strictly signal through one signaling pathway. Therefore, our study could be, and most likely is, overlooking various facets that resulted in modulation of the cachectic phenotype. Ovarian cancers are known to induce expression of TNFα and IL-1β [39]. Upstream of the IKK complex, TNFα and IL-1β signaling converges on the molecule TAK1 [47]. In addition to activating canonical NF-κB signaling, TAK1 activates the p38, JNK, and ERK MAPK pathways, all of which can influence skeletal muscle atrophy [47]. Further distal of the NF-κB pathway than what was explored in the present study are the ubiquitin proteasome system and autophagy signaling pathways [48, 49]. Proteolysis is orchestrated through these signaling paradigms and upregulation is frequently evidenced in cachectic states [48, 49]. We plan to follow-up the present study with an exploration further downstream of the signaling pathway investigated herein and the ramifications on cachectic signaling and whether or not WFA affects these signaling pathways.

From our results, we conclude that WFA is a novel mediator of upstream cachectic signaling in our xenografted ovarian cancer model. Future work on WFA could pave the way for a novel therapeutic treatment of cancer-induced cachexia after deeper investigations into the downstream mechanistic pathways of WFA. A limitation of the study was the cell line of choice used, as recent works have demonstrated that the A2780 cell line is more genetically similar to the relatively rarer endometroid ovarian cancers [50, 51] than high-grade serous ovarian cancer, which accounts for 70% of ovarian carcinomas [52]. The cell line was chosen for its ability to readily xenograft and rapidly develop tumors [53], theoretically inducing a cachectic phenotype at an accelerated rate. It remains to be seen whether a more clinically relevant cell line that is more genetically similar to high-grade serous ovarian cancer [54], such as the obscure KURAMOCHI cell line [50], will induce a cachectic phenotype and if WFA can has a significant effect on proinflammatory cytokines produced from this cell line.

## Supplementary information

**Additional file 1: Table S1.** Human and mouse gene specific primer sequences.

**Additional file 2: Table S2.** Primary and secondary antibody list.

## Data Availability

All data generated or analyzed during this study are included in this published article and its supplementary information files. All raw data/images and quality control reports related to this manuscript are available from the authors. Requirements for external data sets not applicable (none used).
