# Supplementary material for: Withaferin A ameliorates ovarian cancer-induced cachexia and proinflammatory signaling
Source: J Ovarian Res. 2019 Nov 25;12:115. doi: 10.1186/s13048-019-0586-1 (PMC6878639; doi:10.1186/s13048-019-0586-1)
Supplement: Supplementary file 1 — Additional file 1: Table S1. Human and mouse gene specific primer sequences. [file 13048_2019_586_MOESM1_ESM.docx]

**TableS1**: Human and mouse gene specific primer sequences.

| **Gene** | **Species** | **Forward** | **Reverse** |
| --- | --- | --- | --- |
| *TNFα* | Homo sapiens | 5’-CCCAGGGACCTCTCTCTAATC-3’ | 5’-ATGGGCTACAGGCTTGTCACT-3’ |
| *TNFα* | Mus musculus | 5’-AGCACAGAAAGCATGATCCG-3’ | 5’-GCCACAAGCAGGAATGAGAA-3’ |
| *IFNγ* | Homo sapiens | 5’-CTAATTATTCGGTAACTGACTTGA-3’ | 5’-ACAGTTCAGCCATCACTTGGA-3’ |
| *IFNγ* | Mus musculus | 5’-GACAATCAGGCCATCAGCAAC-3’ | 5’-CGGATGAGCTCATTGAATGCTT-3’ |
| *IL-6* | Homo sapiens | 5’-ACACAGACAGCCACTCACCT-3’ | 5’-TTCTGCCAGTGCCTCTTTGC-3’ |
| *IL-6* | Mus musculus | 5’-CCTTCTTGGGACTGATGCTGG-3’ | 5’-GCCTCCGACTTGTGAAGTGGT-3’ |
| *IL-8* | Homo sapiens | 5’-AAACCACCGGAAGGAACCAT-3’ | 5’-CCTTCACACAGAGCTGCAGAAA-3’ |
| *MIP-2* | Mus musculus | 5’-CCACTCTCAAGGGCGGTCAAA-3’ | 5’-TACGATCCAGGCTTCCCGGGT-3’ |
| *GAPDH* | Homo sapiens | 5’-TGATGACATCAAGAAGGTGGT-3’ | 5’-TCCTTGGAGGCCATGTGGGCC-3’ |
| *β-Actin* | Mus musculus | 5’-CAGGCATTGCTGACAGGATG-3’ | 5’-TGCTGATCCACATCTGCTGG-3’ |
