# Supplementary material for: Withaferin A ameliorates ovarian cancer-induced cachexia and proinflammatory signaling
Source: J Ovarian Res. 2019 Nov 25;12:115. doi: 10.1186/s13048-019-0586-1 (PMC6878639; doi:10.1186/s13048-019-0586-1)
Supplement: Supplementary file 2 — Additional file 2: Table S2. Primary and secondary antibody list. [file 13048_2019_586_MOESM2_ESM.docx]

**Table S2:** Primary and secondary antibody list.

| **Antibody** | **Company** | **Catalog #** | **Application** | **Dilution** |
| --- | --- | --- | --- | --- |
| Monoclonal anti-MyHC Type I | Developmental Studies  Hybridoma Bank | BA-D5 | IHC | 1:100 |
| Monoclonal anti-MyHC Type IIa | Developmental Studies  Hybridoma Bank | SC-71 | IHC | 1:100 |
| Monoclonal anti-MyHC Type IIb | Developmental Studies  Hybridoma Bank | BF-F3 | IHC | 1:100 |
| Polyclonal anti-phospho-RelA (pSer^536^) | Sigma-Aldrich | SAB4300009 | IHC | 1:50 |
| Alexa Fluor™ 488 Goat anti-Mouse IgM (μ) | Thermo Fisher Scientific | A-21042 | IHC | 1:2000 |
| CF™ 568 Goat anti-Mouse IgG1 (γ1) | Sigma-Aldrich | SAB4600313 | IHC | 1:2000 |
| CF™ 350 Goat anti-Mouse IgG2b (γ2b) | Sigma-Aldrich | SAB4600228 | IHC | 1:2000 |
| Alexa Fluor™ 594 Goat anti-Rabbit IgG (H+L) | Thermo Fisher Scientific | A-11012 | IHC | 1:2000 |

IHC = Immunohistochemistry.
